# Current and Novel Therapies Against Helminthic Infections: The Potential of Antioxidants Combined with Drugs

**DOI:** 10.3390/biom10030350

**Published:** 2020-02-25

**Authors:** Nuno Vale, Maria João Gouveia, Fátima Gärtner

**Affiliations:** 1 Laboratory of Pharmacology, Department of Drug Sciences, Faculty of Pharmacy, University of Porto, Rua de Jorge Viterbo Ferreira 228, 4050-313 Porto, Portugal; 2 i3S, Instituto de Investigação e Inovação em Saúde, University of Porto, Rua Alfredo Allen 208, 4200-135 Porto, Portugal; fgartner@ipatimup.pt; 3 Institute of Molecular Pathology and Immunology of the University of Porto (IPATIMUP), Rua Júlio Amaral de Carvalho 45, 4200-135 Porto, Portugal; 4 Department of Molecular Pathology and Immunology, Institute of Biomedical Sciences Abel Salazar (ICBAS), University of Porto, Rua de Jorge Viterbo Ferreira 228, 4050-313 Porto, Portugal; mariajoaogouveia@gmail.com; 5 Center for the Study in Animal Science (CECA/ICETA), University of Porto, Rua de D. Manuel II, Apt 55142, 4051-401 Porto, Portugal

**Keywords:** schistosomiasis, opisthorchiasis, combined therapy, drug repurposing, anthelmintic drugs, antioxidants

## Abstract

Infections caused by *Schistosoma haematobium* and *Opisthorchis viverrini* are classified as Group 1 biological carcinogen and it has been postulated that parasites produce oxysterol and estrogen-like metabolites that might be considered as initiators of infection-associated carcinogenesis. Chemotherapy for these helminthic infections relies on a single drug, praziquantel, (PZQ) that mainly targets the parasite. Additionally, PZQ has some major drawbacks as inefficacy against juvenile form and alone it is not capable to counteract pathologies associated to infections or prevent carcinogenesis. There is an urgent need to develop novel therapeutic approaches that not only target the parasite but also improve the pathologies associated to infection, and ultimately, counteract or/and prevent the carcinogenesis processes. Repurposing the drug in combination of compounds with different modes of action is a promising strategy to find novel therapeutics approaches against these helminthic infections and its pathologies. Here, we emphasized that using antioxidants either alone or combined with anthelmintic drugs could ameliorate tissue damage, infection-associated complications, moreover, could prevent the development of cancer associated to infections. Hence, antioxidants represent a potential adjuvant approach during treatment to reduce morbidity and mortality. Despite the success of some strategies, there is a long way to go to implement novel therapies for schistosomiasis.

## 1. Helminthic Infections: An Overview

**Figure 1:**
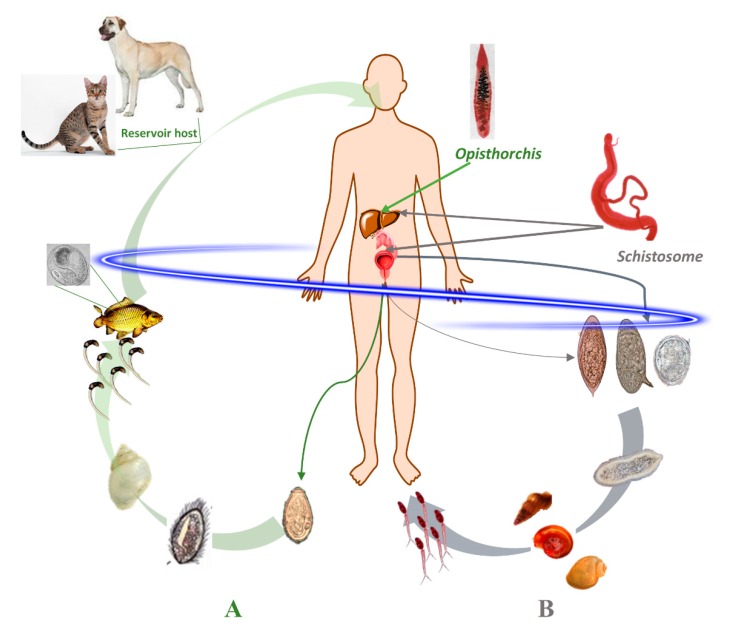
Life cycle of schistosomes (grey) and opisthorchiids (green). Both parasites have a complex life cycle involving two or more hosts. (**A**) The infection with *Opisthorchis* spp. occurs through ingestion of raw fish which contain metacercariae. Following ingestion, the metacercariae excyst in the duodenum and juveniles migrate into the biliary tract where they mature and lay eggs that are excreted through feces. Within the snail, the parasite undergoes an asexual reproduction phase which, in turn, produces the cercariae that are shed from the snail into the water, where they seek out and infect the fish. (**B**) Regarding schistosomes, the infection follows exposure of human skin to contact with water containing the cercariae. These larvae penetrate the skin, shed the tail in the dermis, and transform into the schistosomulum stage which migrates in the circulation. After several weeks, the adult schistosomes take up residence in the venous blood of the intestines or pelvic organs. The adult worms’ mate and proceed to release eggs that are excreted. The eggs hatch on contact with fresh water, releasing miracidia that infect suitable snails, and thereby complete the developmental cycle.

One-third of the global population is estimated to be infected with helminths; hence they are among the most prevalent infectious disease agents, and these infections remain a persistent public health problem in the developing world [1]. Most helminth infections, if left untreated, progress to a chronic inflammatory disorder that caused both concurrent and delayed-onset pathology [2,3]. About a billion people in developing regions of Sub-Saharan Africa, Asia and the America are infected with one or more helminth [4]. Some of the most important helminthiases are caused by food borne trematodes including species of *Opisthorchis* as well as schistosomes [4,5,6]. The International Agency for Research on Cancer (IARC) recognizes infection with *Opisthorchis viverrini*, *Clonorchis sinensis* and *Schistosoma haematobium* as a definitive risk of cancer [7]. In addition, to direct detriment on development and health of infected populations, infections with these parasites frequently lead to development of cholangiocarcinoma (CCA, bile duct cancer) and squamous cell carcinoma of the bladder (SCC) [5]. On following sections, we review the geographical distribution of parasites, its life cycles (Figure 1) and major dire complications caused by their infection.

### 1.1. Schistosomes: Geographical Distribution, Life Cycle and Infection

Three main species of schistosome species are responsible for human schistosomiasis, *Schistosoma mansoni*, *Schistosoma japonicum* cause intestinal schistosomiasis in East Asia, Africa, South America and the Caribbean, while Schistosoma haematobium occurs through Africa and the Middle East, causing urogenital schistosomiasis (UGS) [1,8]. Notably, infection with *S. haematobium* is classified as a group 1 carcinogen [7]. Infection follows exposure to freshwater containing free-swimming larval forms of the parasite which penetrate the human skin. Following penetration, the cercaria loses its tail to become the schistosomulum stage. This developmental stage enters the bloodstream where it circulates for several weeks before the new adult schistosome takes up residence within the mesenteric veins (*S. mansoni* and *S. japonicum*) or the vesicle plexus and veins that drain the ureter and nearby pelvic organs (*S. haematobium*). The female and male worms’ pair, and release eggs. The eggs must transverse the walls of the blood vessel in order to reach the lumen of the intestine or bladder to be excreted. Nonetheless, many eggs become trapped in the tissues or organs where they provoke inflammation and circumoval granuloma formation [6].

Recent outbreaks of *S. haematobium* infection have been reported in Western Europe [9,10,11]. Some authors suggest that hybridization of *S. haematobium* and *S. bovis* has occurred in Corsica. This could increase the range of potential vectors increasing the risk of dissemination to Portugal, Spain and Italy [9,12]. More than 100 million people are infected with *S. haematobium*, more cases than with other schistosomes. Many cases of UGS result ostensibly in only mild symptoms and disease, such as hematuria, dysuria, anemia and inflammation of genital–urinary tract [13,14]. Nonetheless, cases of UGS experience moderate to severe morbidity that ultimately may be followed by squamous cell carcinoma [13,15], which could be related to deposition of S. haematobium parasite ova [16,17]. Bladder cancer is a frequent and dire complication of chronic UGS. Patients with schistosomiasis may develop bladder cancer earlier than uninfected people. The severity and frequency of the sequelae of UGS and its complication are related to the intensity and duration of the infection [18,19].

### 1.2. Opisthorchis: Geographical Distribution, Life Cycle and Infection

The infection is caused by a triad of phylogenetically closely related trematodes—*O. viverrini*, *O. felineus* and *C. sinensis*, and is a major public health problem burden in East Asia, Eurasia and central Europe, affecting more than 45 million people [4,20,21]. All three trematodes have a three-host life cycle with the first intermediate hosts being freshwater snails, the second fish mostly often belonging to the family Cyprinidae, while mammals, in most cases, carnivores and humans, serve as a definitive host (Figure 1) [22]. Although *O. felineus* is not considered a carcinogenic agent as *O. viverrini*, recently, biochemical and histopathological data suggest that it might fit in that pattern [23,24,25].

Opisthorchiasis results from ingestion of the metacercarial stage of parasites encysted in undercooked, freshwater cyprinoid fish. After ingestion of metacercariae, the larvae excyst in the duodenum and migrate through the ampulla of Vater into bile ducts, where they mature into hermaphroditic adult worms that release eggs, which in turn pass out with the bile to the bowel and to the environment with the fecal stream. Freshwater snails ingest the eggs, after which they undergo asexual reproduction until aquatic cercariae that are released to the freshwater and penetrate the flesh of fishes (secondary intermediate hosts) (Figure 1) [26]. The infection is associated with hepatobiliary morbidity, involving cholangitis, obstructive jaundice, hepatomegaly, cholecystitis and/or cholelithiasis [27,28]. The liver flukes cause mechanical injury to the bile ducts, and their metabolic products irritate the biliary epithelial cells, leading to cell desquamation, hyperplasia, dysplasia and eventual fibrosis [26,27]. Importantly, both experimental and epidemiological evidence strongly implicate the liver fluke infection in the etiology of cholangiocarcinoma-bile duct cancer (CCA), a generally fatal cancer [26,29,30,31], related to the difficulty in early diagnosis due to the silent character. Moreover, the therapeutic approaches are scarce and limited, especially in resource poor settings [32].

Although the infections with *S. haematobium* and *O. viverrini* are classified as a Group 1 biological carcinogen [5], much of the cellular and/or molecular mechanisms linking parasitic infections with carcinogenesis remains unclear [33]. Over recent years, our research group has undertaken studies aiming to clarify the role of these infections in helminth infection-associated carcinogenesis [23,34,35,36].

## 2. Parasites and Its Metabolites: Their Role on Pathogenesis and Carcinogenesis Associated to Infection

Carcinogenesis is a complex and multifactorial process. Many multiple factors could trigger the development of cancer associated to infections caused by parasites as spillover effects from local and systemic chronic inflammation (reactive oxygen species, reactive nitrogen species) directed against the worms, the secretion of mitogens and other mediators by the parasite [26], and interactions or changes in the biliary, GI tract and urinary tract microbiota, including by other potentially oncogenic biological species [37], the role of nitrosamines [38,39,40,41]. The concept of chemical carcinogenesis provides insight into the comprehension of SCC emergence in the bladder of humans with *S. haematobium* infection [35,42] and, a similar process may occur in CCA associated to *O. viverrini* infection [24,34]. Several decades ago, Miller and Miller (1981) developed the concept, theory consolidation and principles concerning ultimate carcinogens as strong electrophilic reactants with macromolecules, such as DNA. According to this concept, environmental factors play a strong role in determining the occurrence of many human cancers. These factors may involve the three general classes of carcinogenic agents: certain radiation, virus pathogens, chemicals and combination of thereof [43]. More recently, Cavalieri and Rogan and collaborators emphasized that estrogenic compounds can initiate cancer by reacting with DNA and highlighted specific metabolites of endogenous estrogens such as catechol estrogens-3,4-quinones as reactive with DNA and able to form depurinating estrogen-DNA adducts. Subsequent liberation of these adducts leaves apurinic lesions in the DNA, generating mutations that may initiate breast and other cancers [44,45,46].

Similar metabolites have been detected either in eggs of *S. haematobium* and in biofluids, including serum and urine during UGS [35,47,48]. Furthermore, evidence of interaction of catechol-estrogens quinones (CEQ) with host DNA leading to formation of DNA adducts has been reported in humans during UGS with or without associated bladder cancer [35]. These findings support the notion that these reactive metabolites of estrogens could be mutagens and initiate UGS-induced SCC. In addition, evidence of oxidation of host DNA was also detected in urine during UGS [35]. Histopathological studies revealed that p53 was altered during *S. haematobium* infection and associated bladder cancer, which could be a result of interactions of reactive metabolites from the schistosome [49].

Metabolites of estrogen including catechol-estrogens have been characterized in *O. viverrini* liver flukes from experimentally infected hamsters [34]. Many of the metabolites were oxysterols-like metabolites, which are oxidation products of cholesterol that can be mutagenic or genotoxic, and possess pro-oxidative and pro-inflammation properties to promote carcinogenesis [34]. These kinds of metabolites were also observed in developmental stages of *O. felineus* and biofluids from infected hamsters [23]. In addition, immunohistochemistry studies of hamsters-infected with *O. felineus* demonstrated that infections induce biliary intraepithelial (BilIn) lesion of grade 3, suggesting the presence of pre-cancerous niche. Taken together, these reports indicate that infection of *O. felineus* might be carcinogenic as *O. viverrini*, at least in the rodent model [23]. It remains unclear how and why parasite needs/uses these metabolites; their formation might be related to the physiology of the worms and/or parasite–host interactions that modulate metabolic pathways of steroid hormones and bile acids.

**Figure 2:**
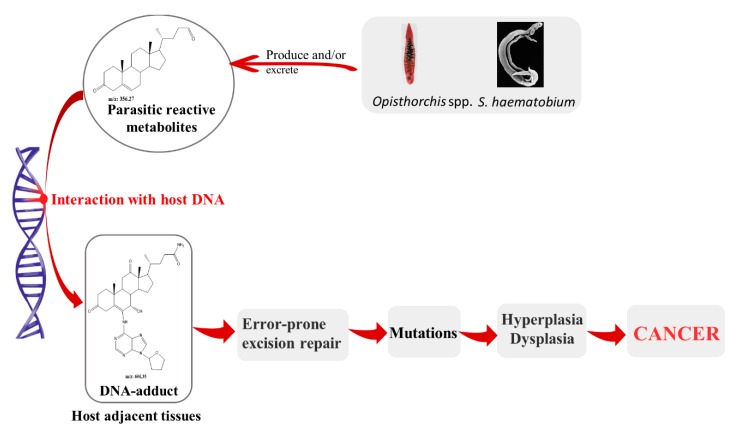
Carcinogenesis mediated by reactive metabolites of *S. haematobium* and *O. viverrini*. Reactive metabolites of parasite origin likely interact with host DNA inducing DNA apurinic sites that may escape the DNA repair mechanisms leading to mutations. These mutations may ultimately transform the target cell, leading to dysplasia and malignant neoplasia.

In view of these considerations, we postulated a potential mechanism involving parasitic reactive metabolites and their interaction with host DNA. This interaction results in lesions in chromosomes and production of depurinating estrogen-DNA adducts leading to parasite metabolite-promoted host cell DNA damage, due to parasite-derived, reactive oxysterol and/or catechol estrogen derivatives. Oxysterols and/or catechol estrogens of trematode origin and/or precursors modified as the consequence of opisthorchiasis or UGS are candidate initiators given that these metabolites mutate genes in other settings. We speculate that this interaction triggers a cascade of events that culminate in development of cancers associated to infection. The formation of DNA adducts leads to apurinic sites that if they were not repaired through an error-prone excision, could lead to mutations and ultimately to cancer (Figure 2) [37,50].

Current treatments for these helminthic diseases mostly target the parasite and not the pathologies associated to infection. In the next section, we will discuss alternative therapies against these diseases.

## 3. Chemotherapy Against Schistosomiasis and Opisthorchiasis

Nowadays, praziquantel (PZQ) is the ‘drug of choice’ for the treatment of opisthorchiasis, schistosomiasis and other diseases caused by trematodes [51,52]. After establishing the evidence base that PZQ is safe and highly effective against all major human schistosome species [53,54], clinical trials were launched in Asia to assess the efficacy of this drug on major foodborne trematodes as opisthorchiasis [55,56]. Thereafter, PZQ was used on mass drug administration (MDA) programs in endemic regions as ‘preventive chemotherapy’ against these parasitic diseases [51]. The success of MDA in reducing the human prevalence of infections, and preventing transmission, depends on many factors, such as the treatment coverage rate, the frequency of treatment campaigns and compliance of treatment [57]. Since 2006, many millions of doses of PZQ have been consumed and it has been estimated that by 2018 as many as 235 million people will be treated with PZQ, only against schistosomiasis [58]. Regarding opisthorchiasis, in 2015, an estimated 600,000 individuals were reported to be treated for foodborne trematodiasis worldwide [59]. Despite MDA programs, liver and blood fluke infections still remain a major public health concern, and prevalence is increasing in some regions [60]. In addition, multiple reinfection is common, and the infections tend to be chronic [61].

According to the World Health Organization (WHO), the recommended dose of PZQ for opisthorchiasis is 75 mg/kg/day orally, three doses per day for two days, while for schistosomiasis it is 40 mg/kg per day divided in two doses for one day [62]. Some authors have suggested that worms have different susceptibilities to PZQ in geographically separated areas, even if they belong to the same species [63], therefore the dosages need to be adjusted according to where they are given. In general, these treatment schedules are well tolerated with only few mild and transient adverse events as abdominal pain, dizziness, headache, nausea and urticaria [64]. Despite the efficacy against the diseases and its safety profile, PZQ presents some drawbacks, including poor solubility and an extensive metabolism via hydroxylation of the absorbed drugs to inactive metabolites [47,65]. Moreover, PZQ is inefficient against juvenile forms including the schistosomula of *Schistosoma* spp. and *Opisthorchis* spp. newly excysted metacercariae (NEM) [63,66]. Although administration of PZQ might clear infection, PZQ alone cannot prevent or ameliorate infection-induced inflammation and fibrosis, and thus the risk factor for the infection-associated cancer remains following infection [61]. Moreover, administration of PZQ does not prevent the continuous reinfection [61]. Interestingly, some studies in the hamster model of *O. viverrini* infection reported that repeated infection and consequence PZQ treatment can increase the risk of CCA [67,68]. Nonetheless, in humans, the evidence for this outcome is unclear [69].

Despite its wide usage, the mechanism of action of PZQ remains unknown. Nonetheless, it has been postulated that PZQ disrupts Ca^2+^ homeostasis [70,71]. PZQ causes immobilization, spasmic contractions, paralysis of the worm accompanied by tegument damage [72,73], as evidenced as extensive swelling, erosion, vacuolization and peeling [63]. The pathological vacuolization of the tegument cells causes leakage of sugars and amino acids and cell lysis leading to the death of the parasite [72,74]. In addition, the hypothesis that PZQ alters Ca^2+^ channels is supported by studies that employed calcium channels blockers and cytochalasin D [75]. PZQ might be a G-protein-coupled receptor ligand, with PZQ acting as an agonist at the human 5-HT_2_B receptor [76].

Due to our reliance of the single drug for these two major parasitic infections, there is a growing and legitimate concern that resistance to PZQ might evolve [77,78,79]. Thus far, PZQ resistance is not of clinical concern, however, field and experimental isolates (either schistosome or opisthorchiids) exhibiting significantly reduced susceptibility or low cure rates have been described, foreboding the emergence of drug resistance in these parasites [80,81,82,83,84].

Considering all these factors above, there is an urgent need to investigate novel therapeutic approaches to treat opisthorchiasis and schistosomiasis. Nowadays, these investigations are more pronounced in the case of schistosomiasis, while in the case of opisthorchiasis, little research is conducted outside of Thailand, South Korea and Russia. For both schistosomiasis and opisthorchiasis, there are good rodent models that mimic the infection caused in humans [26]. It is possible to conduct several types of studies using these models, for example, for novel therapies including novel drugs or combination of novel compounds, vaccines or immunotherapy, characterization of infection and ultimately cancer associated to infection [26,85,86].

What has been done so far to find alternatives to praziquantel? Extensive efforts have been made through synthesis of derivates of PZQ and evaluation of anthelmintic activity either in vitro or in vivo [79,87]. Unfortunately, these derivates did not present better activity in comparison to the parental drug. Additionally, the promising in vitro activity of candidate drugs does not necessarily indicate that the compound will present good in vivo activity since their pharmacokinetics and metabolic profile are key determinants for in vivo efficacy. Indeed, a potential action in vitro did not translate to impressive killing in vivo [52]. Therefore, it is necessary to develop novel therapeutic approaches. Therapeutic strategies such as drug repurposing and combination of different active agents constitute a promising and efficient tool against these helminthiasis [88]. Drug repurposing is a useful strategy to accelerate the drug development process due to lower costs, reduced risk and decreased time to availability of preclinical data [89]. In studies pioneering the concept, rational combination chemotherapy was developed for tuberculosis and other bacterial infections [90]. Nowadays, its use has been extended for chemotherapy of cancer, acquired immune deficiency syndrome (AIDS) [91] and for malaria [92,93]. The major goals of combination chemotherapy are to minimize and/or to delay the appearance of drug resistance [90,91,92], and to achieve an additive/synergistic effect that could translate in reduced doses of drugs and/or minimized side effects [90]. Ideally for opisthorchiasis and schistosomiasis, the combined drugs would exhibit a divergent mechanism of action of PZQ and/or target the immature parasite to enhance cure and eggs reduction rates as well as pathologies associated with infection and thereby improve the chemotherapy [65].

### 3.1. Drug Repurposing and Combine Treatments for Opisthorchiasis and Schistosomiasis

#### 3.1.1. Schistosomiasis

This topic has been revised [65]. Several classes of pharmacological agents including anthelmintics, antimalarials and anti-inflammatory agents among others, have been evaluated against schistosomiasis.

Oxamniquine (OXA) has been suggested for drug repurposing against schistosomiasis where it was the drug of choice for *S. mansoni* for many decades in Brazil [94]. However, it presents a major drawback since it is only efficacious against *S. mansoni*. Combination therapy with PZQ and OXA has been used since 1980, both in the laboratory and the clinic. Yet, findings with this combination are not clear and need further investigation under strict criteria [95,96,97,98,99].

Antimalarials including artesunate (AS), artemether (ART) and mefloquine (MFQ), which are widely acknowledged for their antimalarial activity [100] also are active against schistosomiasis. Interestingly, these compounds are highly active against juveniles [100], whereas PZQ is only effective against adult worms. Therefore, antimalarials were tested either alone or combined with PZQ and evaluated not only in the laboratory but also on clinical trials. In a recent meta-analysis, antimalarials used in combination with PZQ exhibited the increased cure rates for schistosomiasis [101].

Pharmacological agents as anti-inflammatory, ibuprofen and naproxen were also being evaluated against schistosomiasis mansoni. Although these agents do not exhibit antischistosomal activity, they played a role in amelioration of inflammation, biochemical and histopathological consequences related to the intensity of infection. Administration of these drugs combined with PZQ resulted in improvement of parameters mentioned and a decrease in granuloma diameter [102]. Similarly, antifibrotic agents as β-aminopropionitrile-monofumarate salt and β-aminopropionitrile, combined with PZQ reduced sizes of granulomas, alleviate the host resistance to challenge infection [103,104]. Combinations of these drugs with PZQ achieved better results than monotherapies.

Combination of lipid lowering agents including atorvastatin and injectable contraceptive medroxyprogesterone acetate induced tegumental damage and significantly reduce the total number of *S. haematobium* worms recovered from infected hamsters. Intriguingly, female worms were less susceptible to either drugs alone or combined in comparison to males [105]. A synthetic lipid compound, edelfosine, demonstrated activity against schistosomula of *S. mansoni*, thereby counteracting the major shortcoming of PZQ. In addition, combined regimens of these drugs with PZQ in vivo resulted not only in the elimination of developmental stages but also on histopathological parameters as reduced granuloma size and hepatomegaly. Additionally, they potentiated anti-inflammatory actions and favored resistance to re-infection [106]. The findings of this study encourage the search for pharmacological agents used in other clinical areas. In addition, combinations of the different drugs available should be pursued.

Biological and natural agents as antioxidant biomolecules have attracted interest against schistosomiasis. These studies were also extensively reviewed [65]. Several antioxidants were studied, and the results are encouraging, either when administered alone or in combination with other drugs. Nonetheless, clinicals trials to assess the inclusion of antioxidants in therapy against schistosomiasis have yet to be launched. It should be noted that these biological agents are considered pharmacological safe agents [65] and it is expected to induce minimal adverse events.

Most of the antioxidants assessed have shown potential antischistosomal activity either in vitro and in vivo, not only against mature [107,108,109,110,111,112,113,114,115,116] as well as in immature forms of *S. mansoni* and *S. japonicum* [113,117,118,119]. These studies have demonstrated that some antioxidants affect the motor activity of the worm in vitro, revealing a possible perturbation/dysfunction of elements of the neuromuscular system [120]. The neuromuscular systems are a crucial element for schistosomes since they control not only movement, but also the oral and ventral suckers involved with parasite attachment. In addition, they support internal organs including the reproductive, excretory and digestive tracts, and maintenance of the female within the gynecophoral canal of the male [121,122]. It was also observed that antioxidants are capable of inducing severe tegumental alterations [108,111,117,120], which is a crucial organ for protection against host responses, nutrient uptake for parasite development and growth, and plays an important role in host–parasite interaction [123]. Moreover, antioxidants impaired worm coupling [113,114,116,124], a process fundamental to oviposition [107,120,125,126,127,128]. This is a critical issue since the eggs are responsible for the formation of inflammatory granuloma on target organs, and the transmission of disease [128].

Besides the antischistosomal activity, it has been demonstrated that antioxidants are capable of restoring the activity of antioxidant liver enzymes near to the levels detected on controls [107,109,117,119,126,129,130,131]. The increase of antioxidant enzymes activity is usually accompanied by reduction on granuloma size and number, resulting in improvement of the liver architecture and functions [108,125,128,129,132,133]. Another interesting aspect of antioxidants is that they could modulate and immunomodulate response and promote alteration in some cytokines [134,135,136,137,138], which could be also helpful to reduce the size and number of granulomata.

Generally, administration of antioxidants concomitantly with antischistosomal drugs improves not only parasitological but also biochemical parameters [114,126,131,135,136,139]. Therefore, the combined treatment has a dual therapeutic effect and could be related to the fact that both compounds have different modes of action and/or act on different targets. Thus, it is reasonable to hypothesize that the results obtained during these studies are linked to a possible additive/synergistic effect of compounds when administered in a combined regimen. In in vitro studies performed in our laboratory, we have found that the use of antioxidants may potentiate the antischistosomal activity of the drugs. Through TEM studies, it was possible to visualize that the schistosomula incubated with the drug and antioxidant combination had the tegument in disruption unlike those incubated with the compounds alone [140].

Through these studies, we concluded that antioxidants not only present antischistosomal activity per se but also induce amelioration of organ target functions as well as host immunity, at least in model rodents. Clinical trials should be considered in order to verify if similar results obtained in vitro and in vivo are translated to human health. Studies to understand the exact mechanism of action antioxidants are also required. Yet, these encouraging results suggest that antioxidants should be considered as adjuvants in combined treatment of schistosomiasis. Problematically, studies related to the effect of antioxidants against schistosomiasis haematobia are scarce.

#### 3.1.2. Opisthorchiasis

In addition to PZQ, albendazole (ABZ), mebendazole (MBZ) and tribendimidine are available for treatment of clonorchiasis and opisthorchiasis [141,142]. The paucity of alternatives might relate to a certain delay in the studies into systematic biology of opisthorchiids resulting from a relatively lower abundance of the corresponding helminthoses as compared with schistosomiasis and, consequently, insufficient research attention to this problem. Similarities between the morphology, anatomic structure and physiological process typical of trematodes suggest that the label extension and drug repurposing can be successfully applied to development of opisthorchicidal drugs [143].

During the 1980s, in vitro, in vivo and human studies were conducted with ABZ and mebendazole against opisthorchiasis. These drugs have been widely and effectively used in the treatment and control of soil-transmitted nematode infections [53]. However, following the administration of drugs to cases of opisthorchiasis twice daily for 3–4 days, only moderate cure rates were observed, albeit with egg reduction rates of > 92% [144]. Recently, a novel complex of ABZ with polysaccharide arabinogalactan from larch wood, *Larix sibirica* and *Larix gmelinii*, was synthesized and anthelmintic activity against *O. felineus* was evaluated. The arabinogalactan-ABZ complex was highly effective against *O. felineus*, presenting an anthelmintic activity at 10-fold lower doses than the parent drug alone. These complexes also showed lower acute toxicity and hepatotoxicity. The results demonstrated that complexes albendazole:arabinogalactan demonstrated to be safer and more effective than ABZ, suggesting that this could be a possible pathway for the design of novel anthelmintics [145].

As antimalarials show potent activity against schistosomes, they may also have potential for treatment and control of opisthorchiasis. Semi-synthetic artemisinin derivatives as ART and AS were administered at a dose of 400 mg/kg to *O. viverrini*-infected hamsters which resulted in worm burden infections of 78% and 66%, respectively. However, complete elimination of the parasite was not achieved even at a dose of 600 mg/kg and both drugs showed toxicity at a dose ≥400 mg/kg [146]. This could be related to alterations of pharmacokinetic properties of artemisinin in infected hamsters [146]. Therefore, further investigation on the pharmacokinetics of the artemisinin over the course of a liver infection is warranted. Nonetheless, these results are encouraging, and further studies should be performed to understand the mechanism of action of artemisinin against *O. viverrini* and to assess activity on related liver fluke *O. felineus*.

In similar fashion to artemisinin, the appealing antischistosomal properties of MFQ triggered interest in its possible activity against opisthorchiids. A single oral dose of 300 mg/kg of MFQ resulted in high worm burdens not only against juvenile but also against adult *O. viverrini* [147]. The MFQ induces severe tegumental alterations including sloughing, furrowing and blebbling following incubation of *O. viverrini* in vitro, suggesting that MFQ targets the fluke’s tegument. Curiously, in in vivo assays, MFQ displays a slower reaction. The differences between the fast drug action on *O. viverrini* in vitro and the slower reaction in vivo remain elusive but could be related to differences of drug concentrations which were much lower in the hamster bile ducts in comparison to the in vitro concentration [147].

Tribendimidine (TBD), a derivative of amidantel, was developed in an attempt to control tapeworm and threadworm infections endemic in China [148]. Laboratory and clinical investigations demonstrated the therapeutic safety of TBD [148]. Of relevance here, it is active against *Opisthorchis.* The in vivo assays demonstrated that high worm burden reduction was achieved with a single dose of TBD. Exposure of parasites to TBD in vitro at lower drug concentrations lead to its rapid contraction and consequently to death by four hours post-exposure. Similar to MFQ, TBD also induces severe tegumental disruption as sloughing, furrowing and blebbling following administration to *O. viverrini*-infected hamsters. Importantly, damage to the oral sucker of the parasite leads to a complete closure of the mouth of the liver fluke by 48 h post-treatment. However, after 72 h post-treatment, the *O. viverrini* recovered from infected hamsters remain alive in contrast to observed in vitro. The investigators suggested that these differences observed between the fast drug action on *O. viverrini* in vitro and the slower action in vivo might be explained by the extensive biotransformation of the drug. Nonetheless, TBD displays informative trematocidal activity either in vitro and in vivo and the tegument seems to be a potential drug target of this anthelmintic [149]. Recently, two randomized, parallel-group, single-blind, dose-ranging, phase 2 trials in children, adults and adolescent were performed in three *O. viverrini*-endemic villages in southern Laos. Several doses of TBD were evaluated in different ratios according to the age of the children. The aim of this trial was to estimate the dose-response relation in terms of cure rate and egg reduction rate. The results obtained demonstrated that it has excellent efficacy and tolerability at doses of 100 mg/kg and above. Nonetheless, it should be noted that mainly adults and children presented low-intensity *O. viverrini* infection. Thus, further studies including patients with moderate and high intensity are warranted [150]. A combination of PZQ with TBD also was evaluated either in vitro or in vivo. The combination in vivo achieved low to moderate worm burden reductions when both drugs were administered simultaneously or on subsequent days, suggesting antagonistic effects in vivo while in vitro presented a synergistic effect [151]. It remains unclear why this apparent contradiction occurs, but it could be related to pharmacokinetic or pharmacodynamic drug interactions in vivo.

In order to promote new potential drug effects, it is necessary to know potential targets of the parasite. Recently, *O. felineus* cytochrome P450 was shown to be a promising target for the development of therapeutic agents against the disease. This enzyme is active in *O. felineus* tissues and it is crucial for the parasite survival [152,153]. Through analysis in vitro of anthelmintic activity of various CYP inhibitors using standard motility and mortality assays against juveniles and adult *O. felineus*, azole inhibitors were shown to reduce not only CYP activity but also substantially decrease the viability of the liver flukes [154]. The most effective anthelmintic agents against developmental stages in vitro were the antifungal agent miconazole (MCZ) and clotrimazole (CTZ), both approved by the US Food and Drug Administration. The activity of these two agents was comparable to that for PZQ [154]. In addition, combinations of azole substances together with PZQ against juvenile and adult *O. felineus* in vitro and their evaluation in vivo effects of drugs alone or combined with PZQ were performed [151]. Similar findings were seen to those for TBD [147]. The synergistic effect of the PZQ–CTZ and PZQ–MCZ combinations observed in vitro, unfortunately were not evident in vivo [155]. For enhanced efficacy, different dosing ration or schedule may be necessary. The authors considered that low efficacy of these azoles agents could be attributed to low drug concentration in the hepatobiliary system where parasites reside. Repetitive dosing at constant time intervals may maintain appropriate drug levels in the hepatobiliary system [151].

Few other drug candidates with trematocidal properties have emerged over the past few years. Although all compounds mentioned here are marketed drugs, it is necessary to perform clinical trials to confirm their in vivo activity. In contrast to schistosomiasis, there are few studies that evaluate the opisthorchicidal activity of biomolecules agents with an antioxidant profile. Nonetheless, some antioxidant agents were evaluated and achieved informative results not only in elimination of parasite but also in remission of the disease due to infection and even on carcinogenesis. For example, cynaropicrin, a compound of botanical origin, exhibits high anti-*O. felineus* in vivo activity exceeding PZQ efficiency. After treatment, no eggs were recovered, suggesting that the compound totally blocked egg production [156].

The protective effect of melatonin (MEL) against *O. viverrini*-induced oxidative and nitrosative stress and liver injury was investigated in a golden hamster model. MEL was administrated orally in various doses (5 up to 20 mg/kg body weight) for 30 days. The administration of MEL reduces the formation of oxidative and nitrosative DNA lesions in the nucleus of bile duct epithelium and inflammatory cells. In addition, it reduces the mRNA expression of oxidant-generating genes and proinflammatory cytokines (tumour necrosis factor-α (TNF- α)), accompanied by an increase in the expression of antioxidant genes (nuclear erythroid 2-related factor (Nrf2) and manganese superoxide dismutase) [156]. The authors suggested that this antioxidant may be an effective chemopreventive agent against *O. viverrini*-induced CCA. In another study using the same model, the chemopreventive effect of MEL on CCA genesis and liver injury was studied. MEL at 50 mg/kg caused a significant reduction in liver/body weight ratios and decreased tumor volumes and, consequently, increased the survival of animals. In the tumorous tissues, MEL at high dose reduced DNA fragmentation and mitochondrial apoptosis by inducing anti-apoptotic protein in the mitochondrial fraction. Additionally, a high-dose significantly increased mitochondrial antioxidant enzymes and prevented mitochondrial ultrastructural changes in the tumor. The authors considered that MEL, at least, maintained tumor dormancy and moderated the malignancy to a less active form [157]. The combination of *O. viverrini* infection and chemical carcinogen induces CCA in hamsters, likely via inflammation-mediated mechanisms. Therefore, it is reasonable to hypothesize that suppression of inflammatory cells at the initial stages of CCA development would be of benefit. The administration of MEL at dose 50 mg/kg for 30 days exerted an immunomodulatory effect, suppressing eosinophils and Th17 cells and expression of Foxp3. The investigators suggested that MEL may be used for CCA chemoprevention and to reduce liver injury on a rodent model of infection [158]. The combination of this antioxidant with the anthelmintic drug should present a dual mode of action targeting that is not only anti-parasitic but preventative of CCA.

The administration of curcumin (CCM), an antioxidant, to *O. viverrini*-infected hamster reduced oxidative and nitrative DNA damage and the expression of oxidant-generating genes (as iNOS, NK-kB and COX2). On the other hand, it enhanced the expression of antioxidant genes including superoxide dismutases 2 and 3 and catalase. Additionally, administration of CCM lead to amelioration of *O. viverrini*-induced histopathological changes through decreased inflammatory cell infiltration and periductal fibrosis. It was hypothesized that curcumin reduces DNA damage through the suppression of inflammatory responses and balancing of oxidant-antioxidant status [159]. Additionally, long-term treatment with CCM resulted in reduction of periductal fibrosis [160]. The administration of CCM to *O. viverrini*-infected hamsters treated with PZQ revealed that the antioxidant might be an effective chemopreventive agent against oxidative and nitrative stress derived from PZQ treatment during opisthorchiasis via induction of nuclear factor-erythroid 2-related factor 2 (Nfr2) and also induced transcriptional regulation of certain genes that lead to an increase in the level of antioxidant capacity in plasma. In contrast, activity of oxidant genes as nuclear factor-kappa B (NF-κB) was down modulated, leading to a decrease in oxidative/nitrative stress markers and consequently, a reduction in liver injury [161]. Recently, it was demonstrated that nano encapsulated CCM and PZQ were more efficacious than CCM plus PZQ in reducing periductal fibrosis in hamsters. In addition, nano-encapsulated treatment improved morphology of bile canaliculi and prevented alteration of genes involved in bile acid metabolism, which were not seen with CCM alone [162].

The aqueous extract of leaves of the blue trumpet vine, *Thunbergia laurifolia*, a traditional medicine in Asia, when administered to *O. viverrini*-infected hamsters leads to reduction in the aggregation of inflammatory cells surrounding hepatic bile duct and without noteworthy toxic side effects. However, the extract itself did not present any opisthorchicidal activity [163]. A combination of the *T. laurifolia* extract with PZQ reduced inflammatory cell aggregation, and more importantly, inhibited development of CCA. The authors suggested that this inhibition could be correlated to the serum alanine transaminase (ALT) levels, which decreased following administration of extract and/or PZQ, decreasing the liver cell damage. The most promising result was achieved when PZQ treatment was followed by administration of extract leading to possible inhibition of CCA. The reason for this is the decrease in the inflammatory activation after PZQ treatment or immune response from parasite death. Due to its anti-inflammatory effects, *T. laurifolia* may inhibit the host immune response during chronic infection resulting in amelioration of liver pathology and liver function [164]. The combination of anthelmintic activity of PZQ with the anti-inflammatory and antioxidant activity of extract could be useful in the treatment of opisthorchiasis and to retard CCA development.

Administration of xanthohumol (XTH), an antioxidant and anti-inflammatory compound, either alone or in combination with PZQ has effects on DNA damage, reduction status changes including iron accumulation and periductal fibrosis during CCA genesis induced by administration of *O. viverrini* and *N*-dinitrosomethylamine (NDMA) in hamsters. Either alone or combined treatment shows reduction of fibrosis and other markers. However, the DNA damage was markedly reduced when compounds were administered together rather than XTH alone, leading to alteration of redox status and repression of CCA development. Following administration of the combined regime, there was no CCA development; the most severe pathological changes observed in these groups were only bile duct hyperplasia. XTH may repress CCA development via antioxidant activity through protection of cholangiocytes from oxidative stress [165].

In this section, we reviewed the experimental studies in vitro and in vivo as well as human clinical trials involving drug repurposing and anthelmintic drugs alone or combined, summarized in Table 1. In addition, we emphasize the use of active biomolecules as agents with antioxidant properties against schistosomiasis and opisthorchiasis.

## 4. Conclusions

Despite the mass drug administration (MDA) campaigns, schistosomiasis and opisthorchiasis remain major public health problems in endemic regions. It is of concern that these diseases are spreading to Western Europe and other sites. For >40 years, the drug of choice against these two parasitic diseases has been PZQ. Despite its efficacy and safety, it has major shortcomings as alone it cannot resolve the histopathological damage characteristic of chronic infection. Moreover, it does not prevent the carcinogenesis associated with *S. haematobium* and *O. viverrini* infections. Thus, it is necessary to implement novel strategies that ideally act against parasites and target pathologies associated to infection. Several repurposed drugs have been evaluated against these helminthic infections. Antimalarials and tribendimidine achieved notable activity against schistosomiasis and opisthorchiasis, respectively. In addition, several combinations among different agents with PZQ/or other anthelmintic drugs represent encouraging leads for treatment approaches to overcome limitations of PZQ monotherapy. The administration of antioxidants in rodent models of these infections leads to reduction in granuloma and enhances antioxidant and immunological responses to the infections. Moreover, treatment with antioxidants following treatment with PZQ can lead to cessation of cancer development. However, there is a lack of human clinical trials. Nonetheless, novel combinations of anthelmintic drugs with antioxidant biomolecules might provide new avenues for discovering alternatives with dual mode of action against these diseases. Despite many encouraging results as detailed above, few studies have yet focused on the effect of antioxidants against schistosomiasis haematobia, likely related to the difficulty in studying *S. haematobium* in the laboratory. Nevertheless, it is critical that investigations of new therapeutic approaches against this disease are attempted. In the case of opisthorchiasis, there is a robust rodent model of infection but investigation for novel therapeutic approaches is restricted to endemic regions, e.g., Thailand and Russia, because naturally infected fish are the only reliable source of the metacercariae.

**Figure 3:**
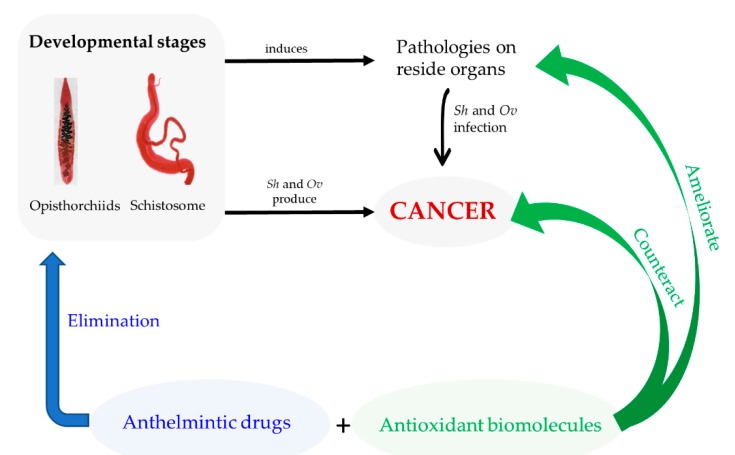
Novel therapeutic approach against schistosomiasis and opisthorchiasis and associated cancers. Through the combination of properties of anthelmintic drugs and biological properties of antioxidants biomolecules, new therapeutic approaches might be developed for anthelmintic therapy and to ameliorate infection induced morbidity. Ultimately, the presence of antioxidants could lead to counteract carcinogenesis through inhibition of the formation of reactive metabolites produced by the parasites.

As noted above, we hypothesized that parasitic reactive metabolites contribute to carcinogenesis initiation through interaction with the host DNA. Some evidence has pointed out that antioxidants can prevent DNA damage and block cancer initiating. Therefore, it is reasonable hypothesized that treatment with antioxidants, either alone or combined, might counteract formation of these parasitic reactive metabolites and ultimately counteract the carcinogenesis. In our point of view, the administration of antioxidants either alone or in combination with other drugs that possess anthelmintic activity could lead not only to amelioration of disease and organ dysfunctions but also might prevent the formation of parasitic reactive metabolites that our research group consider as initiators of the carcinogenesis process associated with infection with *S. haematobium* and liver flukes (Figure 3). Nevertheless, further information related to the effect of antioxidant in counteracting formation of these parasitic reactive metabolites is required.

## Figures and Tables

**Table 1:** Drugs and antioxidants evaluated against schistosomes and opisthorchiids.

Drugs/AntiOx	Model	Treatment	Main Findings	Ref.
Oxamniquine (OXA)	*S. mansoni*-infected mice	OXA plus PZQ	The combinations of the two drugs were markedly superior than those alone.	[95]
		1/3 the curative dose of PZQ plus 1/3 the curative dose of OXA	A potentiating effect was observed in animals receiving combination therapy; Reduction of worm burden and tissue egg load.	[96]
	schistosomiasis mansoni (different parasitic strains: two Venezuelan (YT and SM) and one Brazilian (BH) strain in vivo	Single oral doses of PZQ (250 or 500 mg/kg), oxamniquine (OXA; 40, 60 or 100 mg/kg) or to low-dose combinations of both drugs (33 mg/kg PZQ and 25 mg/kg OXA; 66 mg/kg Pz and 12.5 mg/kg OXA; 250 mg/kg PZQ and 40 mg/kg OXA),	At lower doses of either drug, adult worms of the SM isolate were less susceptible than those of the BH and YT isolates; Lower doses, PZQ more effective in reducing liver or intestinal egg counts than OXA; Males more susceptible to OXA than females.	[97]
	schistosomiasis mansoni and hematobia clinical trial	OXA (4–10 mg/kg) plus PZQ (10-20 mg/kg),	High efficacy of combined regimen in low single doses of 7.5 and 15.0 mg/kg of OXA and PZQ, respectively.	[98]
Artemisinin’s	schistosomiasis mansoni and hematobia In vitro, in vivo and clinical	Alone or combine with PZQ. (review in [65,100,101])	Higher worm burden reductions following treatment with combined regimen compared to PZQ or Artemether alone in vivo; Artemisinin’s highly active against juvenile stage of parasites; Antimalarials used in combination with PZQ exhibited the increased cure rates for schistosomiasis.	[100] [101]
	opisthorchiasis viverrini in vivo	ART and AS were administered at a dose of 400 mg/kg and 600 mg/kg	Worm burden infections of 78% and 66%; complete elimination of the parasite was not achieved at higher dose; Showed toxicity above 400 mg/kg.	[147]
ibuprofen and naproxen	*S. mansoni*- infected mice	alone (200 mg/kg for two weeks) or combine same dosage + PZQ (2 × 500 mg/kg)	Alone did not significantly reduce the worm distribution, egg load or change the program pattern; However, was reduced the granuloma size; Combination ibuprofen and naproxen with PZQ caused a slight increase of percentage of dead ova; marked reduction in the mean granuloma diameter and circulating antigen which was more pronounced than with anti-inflammatory alone.	[102]
β-aminopropionitrile- -monofumara-te salt β-aminopropi-onitrile	*S. mansoni*- infected mice	Alone (5 mg powder of salts in 0.5 mL saline) or combined with PZQ (500 mg/kg b. w.)	Reduced sizes of granulomas and alleviated the host resistance to challenge infection; Decreased liver and spleen weights and a significant reduction in the number of eggs trapped in both liver (86%) and the intestine (99.1%) in comparison to PZQ alone.	[103] [104]
atorvastatin (AV) and medroxy- -proges- -terone acetate (MPA)	*S. haematobium*- infected hamsters	MPA was administered intramuscularly (0.1 mg/kg) at days 7 and 35 p.i. followed by AV treatment regimen (0.9 mg/kg for 49 consecutive days)	Drugs induced tegumental damage and reduced the total number of worms recovered from infected hamsters; Female worms were less susceptible to either drugs alone or combined in comparison to males; Combined regimen decreased the number of eggs in tissue.	[105]
Edelfosine (EDLF)	*S. mansoni* in vitro and in vivo	In vitro: 10 and 20 μM EDLF; In vivo: PZQ (100 mg/kg/day) plus EDLF (45 mg/kg/day) daily 3 days prior to infection until eight days p.i.	In vitro: activity against schistosomula induced interruption of oviposition; In vivo: combination with PZQ resulted not only in the elimination of developmental stages and reduced granuloma size and hepatomegaly; favor resistance to re-infection.	[106]
Albendazole (ABZ)	Opisthorchiasis viverini In vivo	Alone (400 mg twice daily for 3 days)	Moderate cure rates but with egg reduction rates of >92%	[144]
Arabino- .galactan-ABZ complex	Opisthorchiasis felinea in vitro		Anthelmintic activity at 10-fold lower doses than parent drug alone; Lower acute toxicity and hepatotoxicity.	[145]
Mefloquine	Opisthorchiasis viverini In vitro and vivo	Alone (200–400 mg/kg)	High worm burdens not only against juvenile but also against adult worms; Severe tegumental alterations.	[147]
Tribendimidine (TBD)	Opisthorchiasis viverini In vitro, in vivo and clinical trials	In vitro: 0.001, 0.01, 0.1 and 1mg/mL TBD or PZQ. In vivo: Alone (single 400 mg/kg dose) or combined with PZQ (100 and 200 mg/kg)	In vitro: lower drug concentrations lead to its rapid contraction and consequently to death In vivo: high worm burden reduction Combined with PZQ: low to moderate worm burden reductions suggesting antagonistic effects. Clinical trials: excellent efficacy and tolerability at doses of 100 mg/kg and above.	[149] [150] [151]
Miconazole (MCZ) and Clotrimazole (CTZ)	Opisthorchiasis felinea In vitro and in vivo	In vitro: 0.001, 0.01, 0.1, 1, 10, 100 and 500 μM. In vivo: MCZ and CTZ (100 or 200 mg/kg) combined with PZQ (131 or 400 mg/kg b.w.)	In vitro: reduce not only CYP activity and decrease parasites viability; Combined with PZQ: PZQ–CTZ and PZQ–MCZ acts synergistically in vitro but antagonist in vivo.	[154] [155]
*M. armillaris*	*S. mansoni*- infected mice	*M. armillaris* 150 mg/kg orally from 2nd week p.i. twice a week for 6 weeks plus PZQ at 600 mg/kg, orally for 2 consecutive days after 8 weeks p.i..	Combined regimen ameliorated antioxidant enzymes activity and lipid peroxides; Oil enhanced antioxidant system defense ameliorated pathologies associated with infection.	[107]
Resveratrol	*S. mansoni*- infected mice	20 mg/kg once daily for 2 weeks	Ameliorated antioxidant system and lipid metabolism. Significant improvement of specific biomarkers of lung and brain homeostasis.	[109]
	*S. mansoni* in vitro	Alone (100 μM) or combined with PZQ at constant ratio 1:1.	Alone presented moderate activity against schistosomula but combined with PZQ enhanced anthelmintic activity of drug.	[140]
Sylimarin	*S. mansoni*- infected mice	10, 20 or 25 doses of 10 mg/kg Syl at 55 days p.i.	Did not present antischistosomal activity; Diminished the granuloma and fibrosis.	[110]
	*S. mansoni-* infected mice	Alone (750 mg/kg/day) or combine with PZQ (1000 mg/kg)	Alone: Moderate worm burden reduction and ameliorated egg load in liver; Modulation of granuloma size and conservation of hepatic GSH. Combined regime: Improvement of liver function and histopathology. Did not interfere or affect the antischistosomal activity of PZQ. Almost eradicated the presence of adult worms.	[131]
Limonin	*S. mansoni* In vitro and in vivo	Alone in a single dose of 50 or 100 mg/kg on day 21 p.i.; Same dose given on 56 p.i.	In vitro: Antischistosomal activity more pronounced against immature worms than adult; induced tegument alterations; In vivo: Reduction of worm burden more effective at day 21 p.i. than on day 56 p.i. Significant reduction in the hepatic and intestinal tissue egg load; Ameliorated hepatic pathologies.	[117]
α-Lipoic acid (ALA)	*S. mansoni*- infected mice	ALA (single dose 30 mg/kg) combined with PZQ (500 mg/kg) divided into 2 doses 9 weeks p.i.	Combined regimen results in reduction in the worm burden more pronounced in combined regimen, egg count and granuloma size. Recovered the level serum of hepatic enzymes and increased the tissue level of biomarkers of antioxidant function and stress oxidative.	[129]
*B. trimera*	*S. mansoni-* infected mice	24, 48, 91 and 130 µg/mL	Highest concentration presented better antischistosomal activity, reducing motility; Ceased oviposition at sub-lethal concentrations and induced decoupling.	[120]
*4-Hydroxyquinolin-* *-2(1H)-one* *(BDHQ)*	*S. mansoni-* infected mice	Alone at lower or higher dose or for consecutive days;	Active against larval and mature worms; Affected genital systems either males and females.	[126]
		Alone (600 mg/kg) or combine with PZQ (BDHQ 300 mg/kg + PZQ 250 mg/kg)	BDHQ alone or combined resulted in highly significant reduction in total worm burden; reduction of granuloma size more pronounced with combined regimen.	[134]
*A. sativum*	*S. japonicum* In vitro and in vivo	In vitro: 10^−2^ to 10^−6^ (*v*/*v*) concentration. In vivo: Mice pre-treated with garlic and then infected.	Antischistosomal activity against *S. japonicum* against cercariae; Pre-treated with highest concentration lead to total inhibition of infection.	[135]
	*S. mansoni-* infected mice	100 mg/kg body weight from 1 to 7 days p.i., 14 to 21 or 1 to 42 days p.i.	Affected parasite tegument; induced significant worm burden reduction, hepatic and intestinal ova count. Decreased granuloma number and size; Improved immunological parameters.	[111]
*A. sativum* *+* *A. cepa*	*S. mansoni-* infected mice	*A. sativum* or *A. Cepa*: 2 g/100 g body weight daily for 45 consecutive days. PZQ: 500 mg/kg bw on 2 successive days 45 days p.i.	Almost completely eradicated worms, egg load tissue and presence of granulomas. Ameliorated liver architecture and its functions.	[112]
		In vitro: 0.5–5 ppm In vivo: Same regimen as in vitro.	Highly active against all developmental stages of parasites; Induced decoupling; Enhanced host antioxidant system.	[113]
*N. sativa*	*S. mansoni-* infected mice	Alone (2.5 and 5 mL/kg orally) or in combination with PZQ (500 mg/kg for 2 consecutive days)	Alone: Decreased the number and ova of parasites in liver and also reduced number of granulomas. Combined with PZQ: Improved most parameters with most prominent effect was further lowered in dead ova number over that produced by PZQ.	[114]
	*S. mansoni-* infected mice	Alone (0.2 mg/kg alone) or combined with garlic oil (125 mg/kg p.i.) for successive 28 days, starting 1st day p.i.	Compounds alone resulted reduced number of mature eggs while combined regimen resulted in increase of percentage of dead eggs. Combined regimen had more significant effect on serum enzymes (AST and ALP).	[115]
		Alone (0.2 mg/kg of body weight) for 4 weeks starting from 1st day p.i. or combine with Arthemether (single dose 300 mg/kg b.w. follow 49 days p.i) or PZQ (500 mg/kg) for consecutive days.	*N. sativa* either alone or combined with Arthemether or PZQ resulted in improvement of host immunological response stimulating cytokines. Additionally, ameliorated healing process of granulomas lesion.	[136]
*N-*acetyl- -cysteine	*S. mansoni-* infected mice	Alone (200 mg/kg/day on 1st day after infection for acute phase; On 45th for the intermediate; 59 and 75th for chronic stages) or combined with PZQ (100 mg/kg) from 45th to 49th day p.i.).	Antioxidant alone did not present antischistosomal activity; Combined with PZQ: reduced granulomas size and alone NAC was capable to improve liver fibrosis reducing liver damage.	[132]
		Alone (300 mg/kg 5 days a week for 4 weeks) or combine with PZQ (300 mg/kg 7 weeks p.i.)	Combined regimen improved levels of serum enzymes and decreased the total number of worms and consequently decreased liver egg load.	[133]
	*S. mansoni* in vitro	Alone (100 μM) or combined with PZQ at constant ratio 1:1.	NAC did not present significant activity against schistosomula of *S. mansoni* in vitro. When combined with PZQ, slightly improved its antischistosomal activity, was observed.	[140]
Curcumin	*S. mansoni* in vitro	1.56 to 100 μM	Induced decoupling and affected viability of parasite; Affected parasite´s mitochondria and altered oxidative stress parameters increasing oxidative stress that leads to parasite death.	[124]
	*S. mansoni-* infected mice	300 mg/kg bw after one-month p.i., twice a week for 2 months	Affected the fecundity of adult worms, reducing the number of eggs.	[116]
		Total dose 400 mg/kg bw divided into 16 injections	Reduced presence of parasites and eggs on liver; Improved the infection-associated pathologies as granuloma, hepatic enzymes; increased inflammatory response.	[137]
	*O. viverrini-* infected hamster	Alone administered on normal diet to make the final concentration of 1%(*w*/*w*)	Reduced oxidative and nitrative DNA damage; enhanced the expression of antioxidant genes; Decreased inflammatory cell infiltration and periductal fibrosis;	[159] [160]
		CCM (37, 75 and 150 mg/kg body weight) combine with PZQ	In combined regimen, curcumin decreased oxidative and nitrative stress derived from PZQ treatment and reduced liver injury.	[161]
		CCM (0.40g) and PZQ (300 mg/kg body weight for two constitutive days) nanocapsulated	More efficient than combined regimen without nanocapsulation in reducing periductal fibrosis; Also prevented alteration of genes in bile acid metabolism.	[162]
Melatonin	*S. mansoni-* infected mice	Alone (3.35 mg/kg daily) or combined with cercarial antigen preparation or soluble worm antigen preparation (30 μg/mL)	Mel alone did not decrease worm burden while when combined, almost eliminated parasites completely; Ameliorated oxidative stress.	[125]
	*S. mansoni-* infected mice	Alone (10 mg/kg, 2 weeks) following infection	Reduction of granuloma formation and highly protective against pathological changes not only in liver but kidney; Stimulated antioxidative enzymes and mitochondrial oxidative phosphorylation rendered in amelioration of pathologies associated with infection	[130]
	*O. viverrini-* infected hamster	Alone in several doses (5 up to 20 mg/kg body weight) for 30 days	Reduced the formation of oxidative and nitrosative DNA lesions; increased in the expression of antioxidant genes;	[156]
Melatonin	*O. viverrini-* infected hamster	Alone (50 mg/kg)	Significant reduction in liver/body weight ratios, decreased tumor volumes and maintained tumor dormancy which translated in improvement of animal survival. Exerted an immunomodulatory effect and might act as chemopreventive.	[157] [158]
aqueous extract of *Thunbergia laurifolia*	*O. viverrini* -infected hamsters	Alone (100 mg/kg/dose)	Did not present any effect against worms, however, lead to reduction of the aggregation of inflammatory cells.	[163]
		Extract (100 mg/kg/dose) combine with PZQ (400 mg/kg)	Reduced inflammatory cell aggregation and inhibited development of cholangiocarcinoma.	[164]
Xanthumol		Alone (20 μM or 171 mg/B.W./day) or combined with PZQ (single dose of 400 mg/kg)	Either alone or in combination, xanthumol, presented an effect on DNA damage, ameliorated periductal fibrosis. These effects were more pronounced in combined regimen, leading to suppression of development of cholangiocarcinma. This suppression might be related to antioxidant activity of xanthohumol protecting the cholangiocytes.	[165]

b.w. body weight; p.i. post infection.
